# Species From Feces: Order-Wide Identification of Chiroptera From Guano and Other Non-Invasive Genetic Samples

**DOI:** 10.1371/journal.pone.0162342

**Published:** 2016-09-21

**Authors:** Faith M. Walker, Charles H. D. Williamson, Daniel E. Sanchez, Colin J. Sobek, Carol L. Chambers

**Affiliations:** 1 Bat Ecology & Genetics Laboratory, School of Forestry, Northern Arizona University, Flagstaff, Arizona, United States of America; 2 Center for Microbial Genetics and Genomics, Northern Arizona University, Flagstaff, Arizona, United States of America; Università degli Studi di Napoli Federico II, ITALY

## Abstract

Bat guano is a relatively untapped reservoir of information, having great utility as a DNA source because it is often available at roosts even when bats are not and is an easy type of sample to collect from a difficult-to-study mammalian order. Recent advances from microbial community studies in primer design, sequencing, and analysis enable fast, accurate, and cost-effective species identification. Here, we borrow from this discipline to develop an order-wide DNA mini-barcode assay (Species from Feces) based on a segment of the mitochondrial gene cytochrome c oxidase I (COI). The assay works effectively with fecal DNA and is conveniently transferable to low-cost, high-throughput Illumina MiSeq technology that also allows simultaneous pairing with other markers. Our PCR primers target a region of COI that is highly discriminatory among Chiroptera (92% species-level identification of barcoded species), and are sufficiently degenerate to allow hybridization across diverse bat taxa. We successfully validated our system with 54 bat species across both suborders. Despite abundant arthropod prey DNA in guano, our primers were highly specific to bats; no arthropod DNA was detected in thousands of feces run on Sanger and Illumina platforms. The assay is extendable to fecal pellets of unknown age as well as individual and pooled guano, to allow for individual (using singular fecal pellets) and community (using combined pellets collected from across long-term roost sites) analyses. We developed a searchable database (http://nau.edu/CEFNS/Forestry/Research/Bats/Search-Tool/) that allows users to determine the discriminatory capability of our markers for bat species of interest. Our assay has applications worldwide for examining disease impacts on vulnerable species, determining species assemblages within roosts, and assessing the presence of bat species that are vulnerable or facing extinction. The development and analytical pathways are rapid, reliable, and inexpensive, and can be applied to ecology and conservation studies of other taxa.

## Introduction

Bats (order Chiroptera) comprise 20% of all mammals, with over 1,300 extant species [1], and display extraordinary behavioral and morphological diversity [2]. They are increasingly recognized as important bioindicator species as a measure of ecosystem health [3–5], providers of ecosystem services [6], and effective indicators of regions and habitats with conservation value [7]. Yet, at least 16% of bat species are threatened with extinction, owing to human-caused factors such as urbanization, deforestation, invasive species, roost disturbance, and persecution [6, 8], and a high proportion, relative to most mammalian groups, are data deficient for population estimates and basic biology [8, 9].

Because bats are nocturnal and fly (many travel great distances nightly or annually [10, 11]), most are small in size, and many exhibit minimal morphological differences between species, they are notoriously challenging for conservation and management [12–14]. Often taxonomic identification is a central goal of bat studies (e.g., determining species present at a roost or in a region; differentiating similar-appearing species) and is typically achieved by classic morphology, acoustic analysis of echolocation, or genetics [15–17]. Morphological assessment, in many cases, relies on dental, cranial, or baculum features [18], and thus cannot be applied to live bats. Likewise, acoustic monitoring has practical issues (e.g., underrepresenting species with low-intensity calls, difficulty in differentiating among species with similar calls [19]).

DNA in feces has for over 20 years been recognized as a means to identify species and investigate aspects of biology [20]. Many of the challenges inherent to these types of samples such as high levels of PCR inhibitors and poor DNA quality (degraded DNA) and quantity [21] have now been minimized by improved field collection, DNA extraction, and PCR amplification methods [22–24]. For bats, guano is an appealing non-invasive source of DNA because, unlike bats themselves, it is stationary and easy to sample. Several studies have employed bat guano as a DNA source with great success [24–26], and it has been used for discrimination between co-occurring species at regional scales [26–28]. The next step is a transferable DNA barcoding assay for identification of bats globally that can be applied in various contexts and with little optimization.

DNA barcoding has been welcomed to wildlife studies since it was first proposed as a means of species identification [29]. Effective barcoding approaches require that the DNA region be sufficiently variable for species identification, readily amplify by PCR, that reference sequences from known species are available so amplicons from taxonomically unknown samples can be compared, and the absence of introgression. A 658 bp segment of the mitochondrial gene cytochrome c oxidase subunit I (COI) has robust species-level discrimination in Kingdom Animalia, with unique sequences in over 95% of species [30], and is the most represented animal barcode in public web-based reference libraries. A prominent repository of vouchered barcode sequences, the Barcode of Life Data Systems (BOLD v3, www.barcodinglife.org), includes over 2,600,000 COI sequences from 172,000 animal species (as of August 2016). In bats, low intraspecific variation and high species divergence in COI has been demonstrated [31, 32], and the barcode has been used for both species discovery and taxonomic assessment [33–35].

Primers for a broad coverage of mammals (i.e., universal primers) have been developed [36], but a shorter amplicon is required for consistent amplification of DNA from feces because DNA in this genetic source is highly degraded and difficult to extract—a consequence of time, digestion, humidity, microbes, sunlight, and high amounts of PCR inhibitors [22]. Importantly, “DNA mini-barcodes” 100–300 bp in length have the added advantage that they can be scaled up to cost-effective next-generation sequencing platforms [37]. Universal DNA mini-barcode primers have been developed for eukaryotes [38], but researchers report mixed success with these primers for mammals [38–40], and they will amplify prey items in feces of insectivorous bats. DNA mini-barcodes targeting bats have been published [36], and likely are of great utility for ancient and antique tissue from museum collections, but we found them to have low taxon coverage and perform poorly with feces (below).

Barcoding efforts for ecology, conservation, forensics, and biodiversity can draw from microbial community genomics approaches, which employ bioinformatics tools to design primers with high coverage for diverse study taxa while excluding undesired taxa and build reference libraries that quickly and accurately assign taxonomy, all within inexpensive next-generation sequencing environments. With recent exceptions [37, 41], the non-microbial research community overwhelmingly uses traditional primer development tools. While instrumental for many applications, these methodologies and outcomes can be vastly improved where samples are complex and specific yet diverse taxa are targeted. Disciplines involved in human microbial pathogen detection are on the leading edge of barcoding because speed, accuracy, cost-effectiveness, and the ability to detect rare pathogens among high levels of diverse background DNA are of paramount importance [42]. Three advances in these fields are easily transferable. First, PrimerProspector [43], open source software for *de novo* primer design and analysis, can be used to build primers from tens of thousands of target sequences while excluding non-target sequences. Developed for characterization of microbial communities in high throughput sequencing environments, it can be applied to any nucleic acid sequence, and resulting primers be used in any context (Sanger or next-generation sequencing). Second, recent advances in genomic methods for detecting and evaluating microbial pathogens and microbial communities have vastly increased sensitivity, decreased sequencing error rates, and decreased costs [42, 44]. Degraded, complex samples are ideally suited for next-generation amplicon sequencing because targeted amplicons are short and deep sequencing enables detection of underrepresented sequences. Third, methods to build reference libraries and classifiers for taxonomic assignment have matured. Methods such as the naïve Bayesian classifier, originally developed for classifying bacterial 16S rRNA genes [45] and built into the QIIME microbiome analysis pipeline [46], are used to filter unreliable assignments via confidence estimates [47]. This is important because automated assignment methods for large batches of sequences can have undesirable error rates, a result of currently incomplete (missing taxa) DNA barcode reference databases [48, 49].

**Fig 1:**
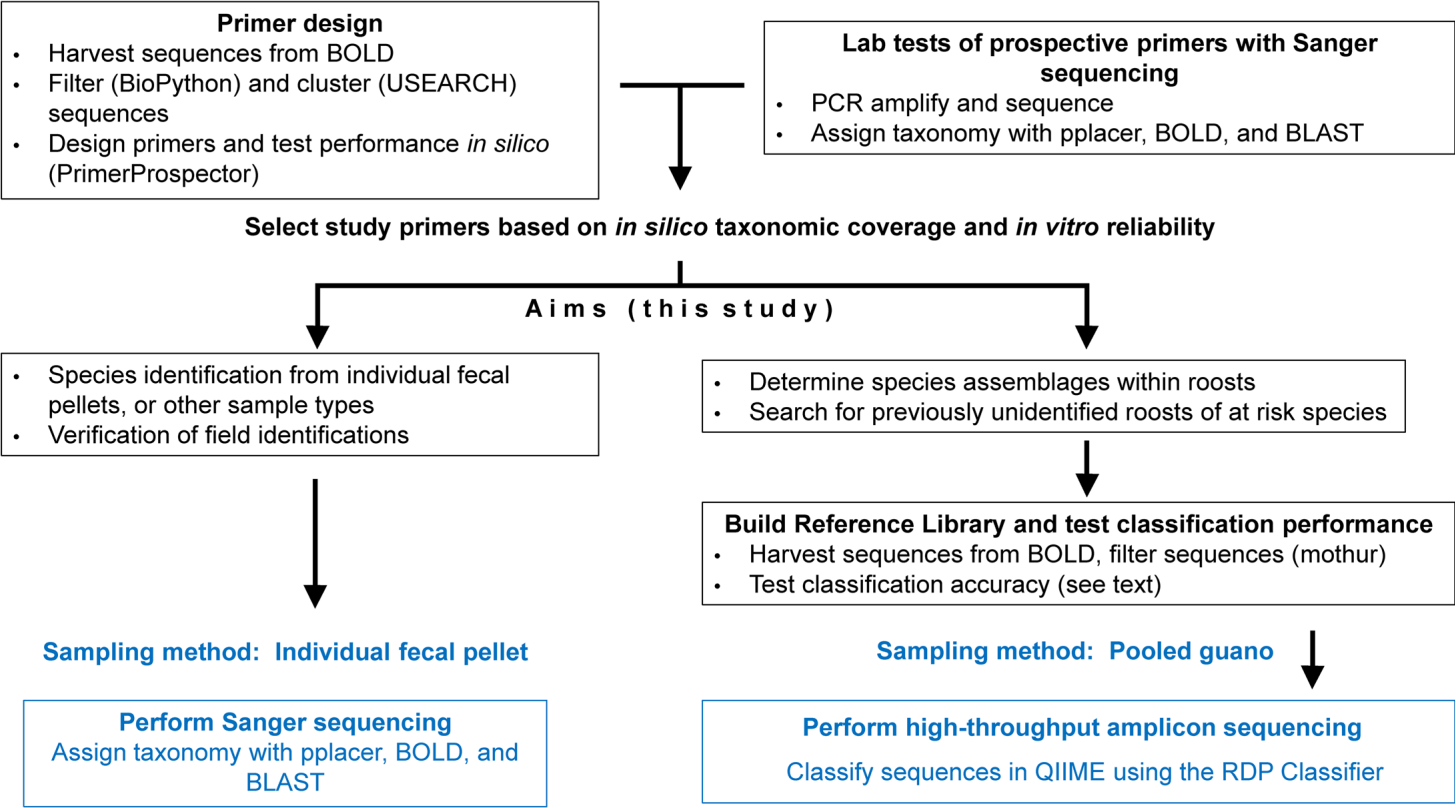
Pathway for Species from Feces assay development. After development, the assay can be applied by following the protocols in blue.

Here, we adopted these approaches for a diverse order of mammals, Chiroptera. Specifically, we identified DNA mini-barcode primers along with a reference library, with the goal of amplifying Chiropteran COI sequences: 1) while excluding COI sequences from non-target taxa that may be present in guano samples (arthropod prey); 2) that were sufficiently short (<300 bp) for reliable amplification of DNA in feces (e.g., aged feces subjected to DNA-degrading conditions), and for use in amplicon sequencing; 3) that perform well for bat species identification globally; and, 4) that can be applied in both traditional Sanger sequencing and cost-effective next-generation sequencing contexts, for a wealth of applications (Fig 1). This study introduces a simple barcoding assay that has high resolution across Chiroptera, and provides an analytical pathway that can be applied to other Eukaryotic taxa that have well-represented databased sequences.

## Materials and Methods

### Taxonomic sampling

#### Genetic collection and storage

We obtained genetic material from 54 species of eight families of bats, including both suborders Yinpterochiroptera (families Pteropodidae, Rhinolophidae, and Rhinopomatidae) and Yangochiroptera (families Molossidae, Mormoopidae, Noctilionidae, Phyllostomidae, and Vespertilionidae). This was achieved by mist-netting bats (feces, buccal swabs) and by loans from collaborators (feces, wing swabs and punches, blood) and museums (internal tissue such as skeletal muscle or heart). Species, numbers, sources, types of genetic material applied for each test are described in relevant sections below. Feces were handled in a Class II type A2 biological safety cabinet (SterilGARD e3, The Baker Company, Sanford, ME, USA), and were processed separately from tissue to avoid cross-contamination. Remaining DNA is cataloged with permanent collection numbers and housed at the Bat Ecology and Genetics Laboratory at Northern Arizona University.

For **primer assessment and validation**, we opportunistically collected buccal swabs (N = 8) and fecal pellets (N = 60) from bats that we mist-netted and visually-identified (AZ: Coconino County, 35.1983 N, 111.6513 W; Navajo County, 36.1336 N, 109.4694 W). Additional samples from collaborators and museums are described below. We used single- or triple-high mist nets (38 mm mesh, 2.6 m x 6-, 9-, 12-, or 18-m net size, Avinet, Inc., Dryden, New York, USA) to capture bats along flyways (river and stream corridors, ponds, forest trails, and unpaved roads [50]). Mist nets were deployed at dusk and remained open for up to 6 hours. For each bat, we recorded species, mass, and forearm length. Buccal cells were collected using the tips of Whatman OmniSwabs (Whatman International Ltd., Maidstone, UK), which were gently rubbed in bat mouths for 1 min, and then ejected into 1.5mL tubes of RNAlater (Ambion, Austin, TX, USA). Fecal pellets were collected singly into 500 μL of RNAlater solution in 1.5 mL tubes. All samples were frozen at -80°C until DNA extraction.

For **next generation amplicon sequencing of pooled guano samples**, we collected a single sample containing approximately 200 (1 gm) fresh-appearing fecal pellets from each of eight abandoned mines used by bats in Arizona (Pima County, 32.489 N, 111.418 W; Mohave County, 35.216 N, 114.362 W), Colorado (Boulder County [two mines], 39.802 N, 105.121 W), New Mexico (Hidalgo County [two mines], 32.171 N, 108.961 W), and Utah (Beaver County [two mines], 38.371 N, 113.06 W). Feces for each sample were deposited into a single 15 mL conical containing 7.5 mL of RNAlater solution, and frozen at -80°C until DNA extraction. RNAlater is a nonhazardous liquid high in salts that stabilizes and protects DNA and RNA [51], is useful for preserving nucleic acids in complex samples such as feces that will be used in several different types of studies (population genetics, parasitology, bacteriology, virology) [52], and is recommended for wildlife studies for which field conditions do not allow immediate freezing [53] or DNA extraction [54] from feces.

#### Ethics statement and permits

Bats were captured and handled using guidelines of the American Society of Mammalogists [55] and with the approval of Northern Arizona University’s Institutional Animal Care and Use Committee (permit numbers 14–008, 07-006-R2, and 15–006), Arizona Game and Fish Department (SP706855), the Navajo Nation (Special Permit 908), and Nicaragua (Autorización de Investigación Cientifica DGPN/DB-IC-009-2015). Captured bats were held for 30 minutes or less, and were released after collection of genetic samples. No bats suffered injury or mortality in this study. Genetic samples of endangered species (*Leptonycteris nivalis*) were obtained from Angelo State Natural History Collections (loan number 2016.01.T).

### Species from Feces: development, coverage, and web tool

#### DNA mini-barcode primer development

We downloaded all COI sequences representing Chiroptera, Arachnida, and Insecta (Taxonomy search terms) from BOLD [56] in April of 2014. Sequences containing ambiguous characters and sequences shorter than 500 nt or longer than 700 nt were removed from the dataset using BioPython scripts [57]. Sequences were then clustered (90% identity for Arachnida and Insecta, 99% identity for Chiroptera) with USEARCH [58]. The resulting dataset (S3 FASTA) was used for primer design and evaluation with PrimerProspector [43]. We selected for lab testing four forward and three reverse primers (Table 1; SFF_145f, SFF_210f, SFF_348f, SFF_351f, SFF_348r, SFF_351r, SFF_492r) designed to produce amplicons of appropriate length.

**Table 1:** Bat COI mini-barcode primers designed and tested in this study. A) Primer sequences; B) PCR amplicon length (bp) and annealing temperature (Ta) for each primer combination. Forward and reverse primers are indicated by f and r, respectively. After lab testing, we selected primer pair SFF_145f/SFF_351r for this study.

A)	COI primer	Sequence (5' - 3')	B)	COI primer	SFF_348r	SFF_351r	SFF_492r
	SFF_145f	GTHACHGCYCAYGCHTTYGTAATAAT		**SFF_145f**	199^a^	202 ^a^	344 ^a^
	SFF_210f	GGAAAYTGRYTARTHCCHYTRATAATTGG		**SFF_210f**	133 ^a^	136 ^a^	278^b^
	SFF_348f	CMGTHTAYCCYCCYYTAGCAGG		**SFF_348f**	**-**	**-**	140^c^
	SFF_351f	CMGTHTAYCCHCCHYTAGCAGGAAA		**SFF_351f**	**-**	**-**	137 ^a^
	SFF_348r	GCATGDGCDAGRTTYCCNGC					
	SFF_351r	CTCCWGCRTGDGCWAGRTTTCC					
	SFF_492r	ACDGATCAKACRAAYARKGGTG					

^a^Ta 56°C

^b^Ta 54°C

^c^Ta 59°C

We also evaluated previously developed DNA mini-barcode primers suggested for forensic barcoding of bats (VF1: [59]; BC1R, BC2F, BC2R, BC3F, BC3R, BC4F, BC4R, BC5F, BC5R, BC6F [36]; VR1: [60]). We used PrimerProspector to predict the primer coverage of Chiroptera and lab-tested the primers with a panel of fecal DNA sources.

#### Primer assessment and Sanger sequencing from single fecal pellets

Samples comprised various tissue types and feces from 54 bat species of the families Molossidae, Mormoopidae, Noctilionidae, Phyllostomidae, Pteropodidae, Rhinolophidae, Rhinopomatidae, and Vespertilionidae (Table 2). We first tested primers on 100 tissue samples (internal tissues, blood, wing punches, buccal swabs, hair and associated cells, wing swabs used for *Pseudogymnoascus destructans* [*Pd*] detection) of 31 bat species to determine whether primers successfully PCR-amplified with various tissue types, whether the products differed between species, and to identify the best performing primer pair combinations. DNA extractions were performed with a DNeasy Blood and Tissue Kit (Qiagen, Valencia, CA, USA) using the Animal Tissue Spin-Column protocol; tissues were subjected to lysis for at least 12 hours. A pre-extraction step was added for buccal swabs in order to remove the salts of RNAlater solution: we centrifuged samples for 10 minutes at 10,000 x g to pellet cells, replaced RNAlater with 500 μL 1X Tris-EDTA (TE), vortexed, and let swabs soak for 1 hour. We repeated the centrifugation step and removed 1X TE before continuing with the Qiagen Blood and Tissue protocol described above.

**Table 2:** Laboratory testing of the Species from Feces mini-barcode assay across sample types of 54 *a priori* identified bat species.

Species	Common name	DNA source	N	Collection number^1^^,^^2^	Taxonomic resolution
*Antrozous pallidus*	Pallid bat	Fecal (10)	10	BEGL:ANPA:G0001-10	Species
*Artibeus jamaicensis*	Jamaican fruit-eating bat	Fecal (1)	1	BEGL:ARJA:G0072	Species
*Carollia perspicillata*	Seba’s short-tailed bat	Fecal (3)	3	BEGL:CAPE:G0069,73–74	Species
*Carollia subrufa*	Gray short-tailed bat	Fecal (2)	2	BEGL:CASU:G0055,61	Species
*Choeronycteris mexicana*	Mexican long-tongued bat	Buccal	5	UA:CHME:B0005-6,8, UCSC:CHME:EB03CM001-2	Species
*Corynorhinus townsendii*	Townsend's big-eared bat	Fecal (1), Wing swab	3	BEGL:COTO:G0011, UNH:Gd11762,11764	Species
*Eidolon helium*	Straw-coloured fruit bat	Fecal (2)	2	OBC:EIHE:G0085, BCI:EIHE:G0086	Species
*Eptesicus fuscus*	Big brown bat	Fecal (8)^3^, Internal tissue, Blood	15	BEGL:EPFU:B0001-2,G0012-19,USDA_APHIS:I0002-6	Species
*Eptesicus furinalis*	Argentine brown bat	Fecal (4)	4	BEGL:EPFUR:G0059-60,66,75	Species
*Euderma maculatum*	Spotted bat	Internal tissue	3	MSB:Mamm:121373, MSB:DGR:22756, NMMNH:EUMA:4059	Species
*Eumops floridanus*	Florida bonneted bat	Fecal (4), Wing punch	6	FFWCC:WP0001-2; BCI:G0020-23	Species
*Eumops glaucinus*	Wagner's bonneted bat	Fecal (1)	1	BEGL:EUGL:G0076	Species
*Eumops perotis*	Greater western mastiff bat	Buccal, Internal tissue	2	BEGL:EUPE:B0003,I0007	Species
*Eumops underwoodi*	Underwood's bonneted bat	Fecal (1)	1	BEGL:EUUN:G0077	Species
*Idionycteris phyllotis*	Allen's big-eared bat	Internal tissue	3	BEGL:IDPH:I0001,I0008-9	Species
*Lasionycteris noctivagans*	Silver-haired bat	Internal tissue, Wing swab	3	BEGL:LANO:I0010, UNH:Gd27811-12	Species
*Lasiurus borealis*	Eastern red bat	Wing swab	2	UNH:Gd26866-67	Species
*Lasiurus blossevillii*	Western red bat	Fecal (1)	1	BEGL:LABL:G0067	Species
*Lasiurus cinereus*	Hoary bat	Internal tissue	7	BEGL:LACI:I0011-17	Species
*Leptonycteris nivalis*	Mexican long-nosed bat	Internal tissue	3	ASNHC17397-9	Species
*Leptonycteris yerbabuenae*	Lesser long-nosed bat	Hair, Buccal, Wing punch	6	BEGL:LEYE:I0043, UA:LEYE:B0007,9–10, UCSC:LEYE:WP201613LY07-6	Species
*Macrotis californicus*	California leaf-nosed bat	Wing punch	1	BEGL:MACA:WP0003	Species
*Myotis auriculus*	Southwestern myotis	Fecal (6)	6	BEGL:MYAU:G0024-28,30	Species
*Myotis californicus*	California myotis	Buccal	1	BEGL:MYCA:B0004	Genus
*Myotis evotis*	Long-eared myotis	Fecal (2)	2	BEGL:MYEV:G0038,39	Genus
*Myotis grisescens*	Gray bat	Wing swab	2	UNH:Gd27724-25	Species
*Myotis lucifugus*	Little brown bat	Internal tissue, Wing swab	6	NYSDEC:MYLU:I0018-21, UNH:Gd24492-93	Species
*Myotis occultus*	Arizona myotis	Fecal (4), Internal tissue	6	BEGL:MYOC:I0022-23,G0034-37	Genus
*Myotis riparius*	Riparian myotis	Fecal (1)	1	BEGL:MYRI:G0068	Species
*Myotis septentrionalis*	Northern long-eared bat	Fecal (2), Wing swab	4	USFS:MYSE:G0090,92, UNH:Gd23912-23913	Species
*Myotis sodalis*	Indiana bat	Wing punch, Wing swab	4	BEGL:MYSO:WP0004-5, UNH:Gd24509-24510	Species
*Myotis thysanodes*	Fringed myotis	Fecal (1)	1	BEGL:MYTH:G0040	Genus
*Myotis velifer*	Cave myotis	Fecal (8), Wing swab	10	BEGL:MYVE:G0041-48, UNH:Gd14953,14955	Species
*Myotis volans*	Long-legged myotis	Fecal (2)	2	BEGL:MYVO:G0089,91	Species
*Myotis yumanensis*	Yuma myotis	Wing swab	1	BEGL:MYYU:WS0001	Species
*Noctilio leporinus*	Greater fishing bat	Fecal (2)	2	BEGL:NOLE:G0078-79	Species
*Nycticeius humeralis*	Evening bat	Wing swab	2	UNH:Gd26834,38	Species
*Nyctinomops macrotis*	Big free-tailed bat	Internal tissue	5	BEGL:NYMA:I0024-28	Species
*Parastrellus hesperus*	Western pipistrelle	Fecal (1)	1	BEGL:PAHE:G0051	Species
*Perimyotis subflavus*	Eastern pipistrelle	Wing swab	2	UNH:Gd24489-24490	Species
*Phyllostomus discolor*	Pale spear-nosed bat	Fecal (1)	1	BEGL:PHDI:G0080	Species
*Pteronotus parnellii*	Common mustached bat	Fecal (3)	3	BEGL:PTPA:G0057,65,81	Species
*Pteronotus personatus*	Lesser mustached bat	Fecal (2)	2	BEGL:PTPE:G0062,82	Species
*Pteropus vampyrus*	Large flying fox	Fecal (1)	1	OBC:PTVA:G0087	Species
*Rhinolophus ferrumequinum*	Greater horseshoe bat	Wing swab	2	UNH:Gd20777-78	Species
*Rhinolophus hipposideros*	Lesser horseshoe bat	Wing swab	2	UNH:Gd29798,835	Species
*Rhinolophus pusillus*	Least horseshoe bat	Wing swab	2	UNH:Gd20779-80	Species
*Rhinopoma cystops*	Egyptian mouse-tailed bat	Wing swab	2	UNH:Gd29880-81	Species
*Rhinopoma microphyllum*	Greater mouse-tailed bat	Wing swab	1	UNH:Gd29895	Species
*Rhogeessa bickhami*	Binkham's little yellow bat	Fecal (4)	4	BEGL:RHBI:G0053-54,83–84	Species
*Rousettus aegyptiacus*	Egyptian fruit bat	Fecal (1), Wing swab	3	UNH:Gd29943-44, OBC:ROAE:G0088	Species
*Sturnira parvidens*	Little yellow-shouldered bat	Fecal (5)	5	BEGL:STPA:G0056,58,64,70,71	Species
*Tadarida brasiliensis*	Mexican free-tailed bat	Fecal (1), Internal tissue	15	BEGL:TABR:I0029-42,G0052	Species
*Tonatia saurophila*	Stripe-headed round-eared bat	Fecal (1)	1	BEGL:TOSA:G0063	Species

^1^ Angelo State Natural History Collections; BEGL: Northern Arizona University's Bat Ecology and Genetics Laboratory; BCI: Bat Conservation International; FFWCC: Florida Fish and Wildlife Conservation Commission; MSB: Museum of Southwestern Biology; NYSDEC: New York State Department of Environmental Conservation; OBC: Organization for Bat Conservation; UA: University of Arizona, UCSC: University of California Santa Cruz; UNH: University of New Hampshire; USDA APHIS: US Department of Agriculture Animal and Plant Health Inspection Service; USFS: US Forest Service Northern Research Station

^2^ See S4 Table for GenBank accession numbers.

^3^ Feces stored at ambient temperature for 3 months.

We then tested primer combinations that exhibited consistently strong amplification and high specificity (single bands on gel) on 86 individual fecal samples of 31 species (Table 2) to assess performance (specificity to the intended DNA region and taxon despite high levels of background DNA and inhibitors, and amplification reliability) with fecal DNA and to validate fecal-derived DNA mini-barcodes for bats with *a priori* (visual) species identification. The validation panel comprised feces from various feeding guilds (insectivores, nectarivores, and frugivores) Fecal samples were fresh (collection methods described above), apart from a subset that were held at room temperature for three months before DNA extraction. We subjected feces to the pre-extraction step described above, and performed DNA extraction with a QiaAmp Fast Stool Mini Kit (Qiagen, Valencia, CA, USA) following the human DNA protocol.

We PCR-amplified the COI regions using the following protocols. PCRs for low quality and quantity DNA from feces, buccal swabs, and wing swabs contained 2 μL undiluted DNA template in a 10 μL reaction, with 1 μL 10X Mg-free PCR buffer (Invitrogen, Thermo Fisher Scientific, Waltham, MA, USA), 2.5 mM MgCl_2_, 0.2 mM of each dNTP, 0.4 μM unlabeled primers, and 0.3 U/ μL PlatinumTaq DNA polymerase (Invitrogen, Thermo Fisher Scientific, Waltham, MA, USA). Cycling involved an initial step of 95°C for 10 min, followed by 38 cycles of 60 s at 95°C, 30 s at 60°C, and 30 s at 72°C, and concluding with a final extension step of 72°C for 10 min. PCRs for DNA from internal tissues and wing punches followed the same protocol except DNA was standardized to 2 ng (DNA quantified with NanoDrop 8000 spectrophotometer, Thermo Fisher Scientific), PlatinumTaq polymerase was lowered to 0.05 U/ μL, and the total number of cycles was reduced to 35.

We used MJ Research PTC-200 thermocyclers for PCRs, and visualized PCR products on 2% agarose gels. At least one negative control was included in all PCRs for this study. PCR products were purified using the ExoSAP-IT product cleanup protocol (Affymetrix, Santa Clara, CA, USA), and were added undiluted to a sequencing reaction using BigDye Terminator v3.1 kit according to the recommended protocol (Applied Biosystems, Foster City, CA, USA). We then sequenced products in both directions on an ABI3130 Genetic Analyzer (Applied Biosystems, Foster City, CA, USA) and edited sequences with Sequencher 5.3 (http://www.genecodes.com), software. Species identity was evaluated using the BOLD identification tool and NCBI’s Basic Local Alignment Search Tool (BLAST; both alignment based), as well as a non-alignment based approach via the program pplacer [61]. This latter method employs maximum-likelihood and Bayesian phylogenetic placement to assign lab-generated sequences onto a fixed reference tree. We screened Sanger-derived sequences for presence of nuclear pseudogenes (NUMTs) [62, 63] by assessing trace files for heterozygous peaks and using a function in BOLD that identifies and flags stop codons.

#### Primer coverage and taxonomic resolution

In an effort to track the utility of our system across Chiroptera, we sought to determine two key parameters for every species: the ability of our primers to successfully hybridize and thus amplify the DNA mini-barcode, and the resolving power (species-level uniqueness) of the DNA mini-barcode. To predict primer coverage, we used PrimerProspector’s *in silico* amplicon generator (get_amplicons_and_reads.py) across all publically accessible Chiroptera COI sequences, combined with our primer combinations as input. This resulted in a FASTA file of those COI sequences trimmed to each mini-barcode.

Species-resolving power of our favored DNA mini-barcode (SFF_145f, SFF_351r) was assessed using BLAST. We compiled all mini-barcodes generated by PrimerProspector and manually trimmed all other COI sequences (Sequencher 5.3; http://www.genecodes.com), resulting in a mini-barcode test panel of 430 bat species. Three outcomes were possible: undisputed identification of the correct species, a reliable alignment to the correct species but also to other genera and/or species, or poor alignment to the top five results of the search. For the two latter outcomes, the sequences were also run through the BOLD identification tool because of the database’s higher species diversity for this marker. These results allowed us to classify species according to whether the DNA mini-barcode could confidently resolve to species or to genus.

#### Species from Feces search tool

To provide the species-specific capability of our assay, as determined by the above exercise, in an accessible framework, we developed an online global database that features the most recent list of bat species and taxonomy (provided by N. Simmons and A. Cirranello, American Museum of Natural History), geographic region, country, and IUCN category (from IUCN search; http://www.iucnredlist.org/search). Capability describes predicted success, based on BLAST and *in vitro* results above, for taxonomic discrimination (species-level, genus-level, and barcode unavailable).

### Many species from many feces: applying NGS to guano

#### Reference library and classification performance

With the goal of moving this system to next-generation sequencing, we created a Chiroptera-specific COI reference library for rapidly and accurately assigning taxonomy to the tens of thousands of reads generated by amplicon sequencing. All sequences associated with Chiroptera (a taxonomy search for “Chiroptera” returned over 20,000 sequences) were downloaded from the BOLD database [56] in January of 2016. Sequences that were not labeled “COI-5P” were not considered, and sequences containing ambiguous characters and sequences shorter than 500 nt were removed from the dataset with mothur (trim.seqs) [64]. We also excluded sequences if they were not identified to the genus or species level. We added three species that had not been previously barcoded for the conventional COI-5P region to the reference dataset: *Macrotus californicus* (GenBank accession number KR337727), *Eumops floridanus* (KR337728, KR337729), and *Eumops perotis* (KX022125). To barcode these species, we developed (with the same computational framework used for mini-barcode primer development) and applied a new primer set (BEGLCOIf: GGYGCYTGAGCHGGWATAGT and BEGLCOIr: ARRATDGGRTCYCCYCCTCC) that targets a 580 bp COI region across Chiroptera (PCR amplification details in S1 Protocol). A taxonomy file indicating the classification of each sequence in the library was generated from data downloaded from the BOLD website.

We tested the efficacy of our library in associating a bat species to a DNA mini-barcode using methods similar to those reported previously [47, 65]. We aligned the reference sequences with clustal-omega [66] and trimmed the sequences to the region of the COI gene that corresponds to the mini-barcode amplicon produced by our newly-designed primers (mothur filter.seqs) [64]. We then classified unique (clustered at 100% identity with USEARCH [58]) amplicon sequences against the full reference database with BLAST [67] and the RDP Classifier developed by Wang, Garrity (45) via QIIME [46]. To test classification performance, we clustered the reference database (USEARCH 100% identity) and randomly subsampled 5% of the clustered reference database sequences (QIIME subsample_fasta.py script). We classified the mini-barcode amplicon region of these subsampled sequences against the remaining 95% of the clustered reference database sequences. Five random subsamples were generated, from which we evaluated the number of correctly classified sequences, the number of false positives (defined here as a sequence incorrectly classified to the taxonomic level under investigation) and the number of false negatives (defined here as a sequence that was not classified to the taxonomic classification level under investigation).

#### Next-generation amplicon sequencing of pooled fecal samples

To assess the feasibility of simultaneously detecting multiple bat species in a collection of guano taken from a roost, we employed the NGS universal tail, dual-indexed amplicon sequencing approach of Colman *et al*. [42] for use with Illumina short-read sequencers. We vortexed each pooled guano sample taken from roosts in abandoned mines (sampling described above) into a semi-slurry, then subsampled 0.22g into a 1.5 mL centrifuge tube and performed the TE soak mentioned above, and then extracted DNA via QiaAmp Fast Stool Mini Kit. Using samples of known multiple species composition, we also tested whether subsampling four times from the same conical resulted in more bat species detected.

We performed a 2-step target-specific PCR on a MJ Research PTC-200 thermocycler, using our optimal unlabeled SFF primer pair (see Results below) in the first reaction followed by a second reaction with incorporated universal tails (UT1 on 5’ end of forward primer: ACCCAACTGAATGGAGC; UT2 to 5’ end of reverse primer: ACGCACTTGACTTGTCTTC). The purpose of the 2-step PCR was to minimize potential primer bias from the incorporated universal tails as well as to prevent extensive primer dimer from solely using the labeled primers, which reduces extraneous purification steps in library preparation, increases robustness of read count, and does not hinder taxonomic recovery. Fecal DNA pools were diluted to 1:5 with molecular grade water and PCR conditions were as described above, aside from an addition of 20 ng non-acetylated bovine serum albumin (Ambion Ultrapure BSA). The second PCR incorporating the UT-labeled primers was scaled up to a 20 μL reaction with 1 μL of undiluted PCR product from the first step, 0.1 U/μL Platinum Taq, and 35 cycles. The resulting amplicons from this initial PCR were employed as template in a subsequent Illumina (MiSeq) extension PCR using unique Illumina indices containing sequences complementary to the universal tails [42].

We removed primer and adapter sequences from Illumina reads with cutadapt 1.6 [68]. Initial sequence processing was performed with commands in mothur [64], and forward and reverse reads were assembled into contigs (make.contigs). We removed sequences containing ambiguous characters, sequences of inappropriate length (screen.seqs), duplicate sequences (unique.seqs), and chimeras (chimera.uchime) from the dataset [64]. Taxonomic classification of sequences was performed in QIIME [46] using the RDP Classifier [45] with a confidence threshold of 0.8. We found this threshold to be an acceptable tradeoff between the number of correctly classified sequences and the risk of false positives (see Results).

As an additional exercise to determine whether our SFF primers have the undesirable capacity to instead PCR amplify DNA of dietary items or arthropods living in the guano, we subjected our pooled fecal samples from the eight mines to custom reference libraries of the four classes of Arthropoda. Sequences and taxonomic information were downloaded from the BOLD database in February 2016 (search terms Insecta, Arachnida, Chilopoda, and Diplopoda), and the requisite FASTA and taxonomy files were created in the same manner as our custom bat reference library and tested with RDP Classifier at 0.8 confidence threshold.

## Results

### Species from Feces: development, coverage, and web tool

#### Primer selection and laboratory validation

Primer pair SFF_145f (GTHACHGCYCAYGCHTTYGTAATAAT) and SFF_351r (CTCCWGCRTGDGCWAGRTTTCC) outperformed the other Species from Feces primer combinations (consistent amplification and single, strong bands), and thus we selected it as our DNA mini-barcode. In laboratory testing with Sanger sequencing, this primer pair reliably (100% success) PCR amplified a single 202 bp region of Chiroptera COI from all sample types (fresh feces and guano at ambient temperature for 3 months, wing and buccal swabs, hair, wing punches, blood, internal tissue) across 54 bat species (known *a priori*) (Table 2), and did not amplify DNA from dietary items or yield extraneous fragments. All sample types generated correct mini-barcode sequences, and all species had sufficiently different, species-specific sequences except for *Myotis thysanodes*/*M*. *evotis* and *Myotis lucifugus*/*M*. *occultus* (S1 FASTA, S4 Table). These species pairs are phylogenetically close relatives within the Nearctic subclade of the monophyletic New World *Myotis* clade [69], and share a mini-barcode as well as standard 658 bp barcode. We found no evidence of nuclear pseudogenes in any of the 186 Sanger-derived sequences.

**Fig 2:**
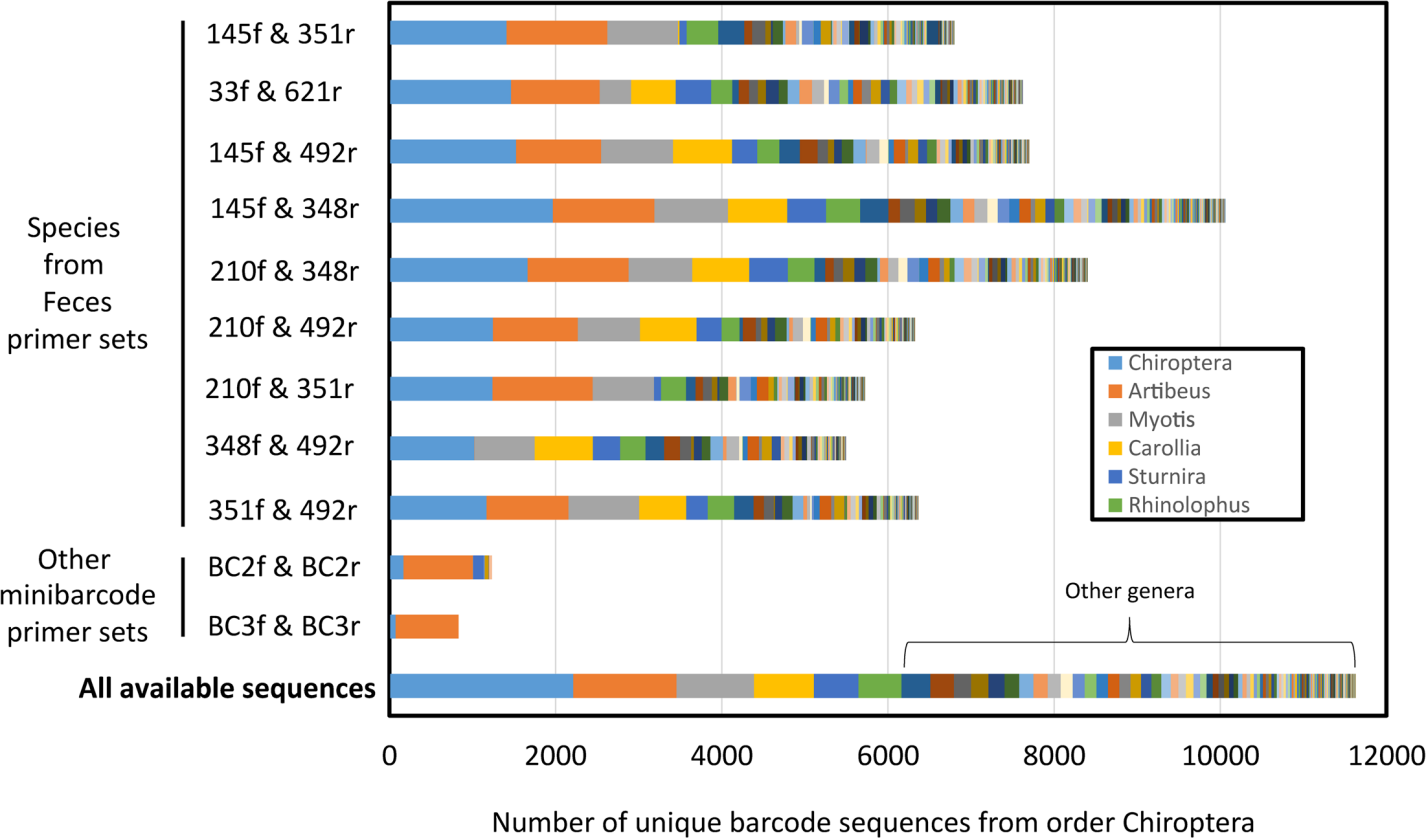
*In silico* taxonomic coverage by genus, comparing bat-specific COI primer pairs developed in the current study, those previously utilized, and all available sequences. We used all publically available barcode sequences for Chiroptera in BOLD and dereplicated them at full length to alleviate taxonomic overrepresentation. Bar components labeled Chiroptera denote bat barcodes from BOLD that were not given taxonomic identifiers below the level of Order.

In silico, our selected Species from Feces primer pair provided considerable coverage (primer affinity to sequences) of bat COI sequence diversity (~58%) out of all available and dereplicated barcodes on BOLD (as of November 2015; > 600 species). All Species from Feces primers outperformed traditionally engineered bat-specific DNA mini-barcodes in number of bat species hit (S1 Table) and taxonomic coverage (Fig 2), and were more capable in the laboratory in terms of reliable amplification of the mini-barcode from fecal derived DNA and resolving to the correct source species (S2 Table).

#### Order-wide Species from Feces barcode resolution

**Fig 3:**
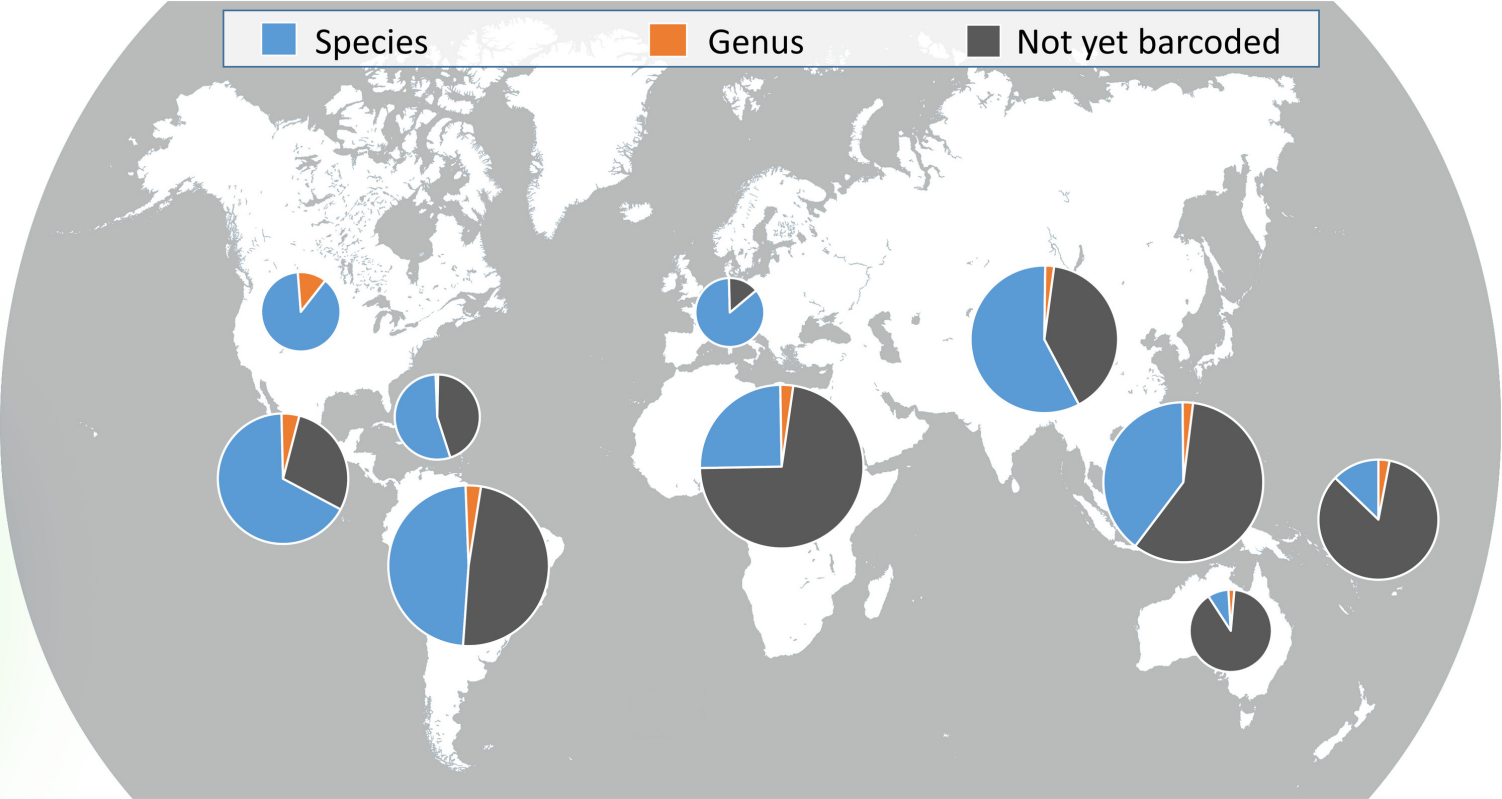
Taxonomic resolution provided by the Species from Feces DNA mini-barcode for bats in each geographic region. Of the species for which COI barcodes are available, 92% can be identified to species, and the remaining share a barcode with other congeners (5%) or across genera (3%). Pie sizes correspond to number of bat species in each geographic region. The dark grey slice includes species not yet barcoded and species for which barcodes are not publically available.

By running our dataset of 430 bat species (S2 FASTA) through NCBI BLAST and the BOLD identification tool for the primer comparison tests, we identified 92% of the dataset correctly to the species level, with the remaining resolvable to genus due to mini-barcode sequence sharing with other taxa (Fig 3). The 36 species found to share a mini-barcode with other taxa were evenly distributed among genera.

#### Species from Feces online: www.nau.edu/batdna

Our Species from Feces database includes all 1,338 known bat species in the most current taxonomy, and can be accessed at http://nau.edu/cefns/forestry/research/bats/search-tool/. Searches can be performed by family, genus, species, region, country, or IUCN Red List category, and a ‘search as you type’ filter is applied for ease of use. Search results indicate whether the Species from Feces mini-barcode is discriminatory to species (452), to genus (31), or whether a barcode is unavailable for the queried species (872). Result lists can be exported, and the database is updated quarterly.

### Many species from many feces: applying NGS to guano

#### Reference library and taxonomic classification performance

**Fig 4:**
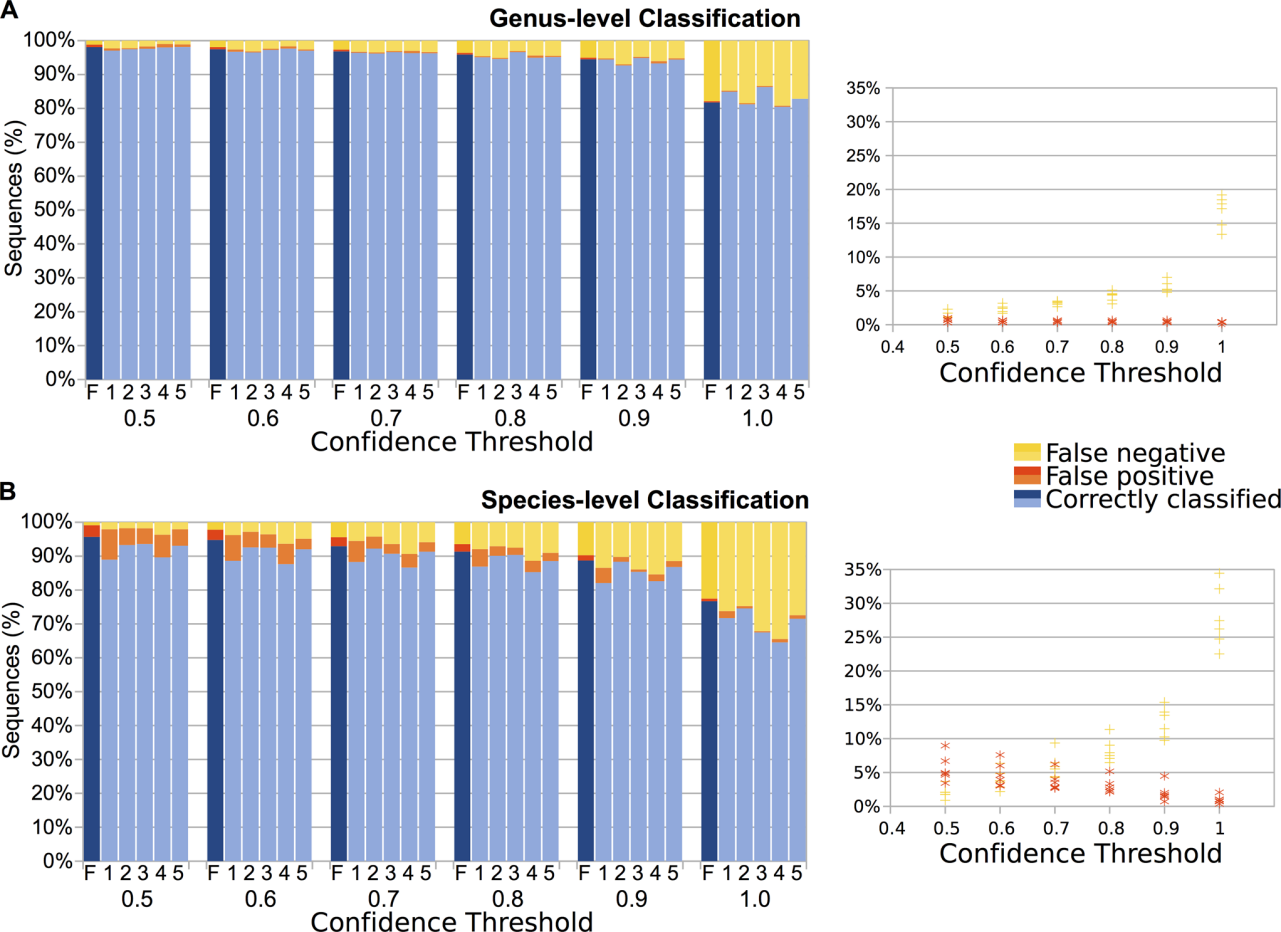
Evaluation of taxonomic classification of reference library sequences. The primer-targeted regions of reference library sequences were classified using the RDP Classifier (in QIIME). We evaluated six datasets: unique amplicon regions against the full reference library (F), and five random subsamples of the reference library (1–5) (see Methods). Classifications with the RDP Classifier were computed with confidence thresholds of 0.5–1.0, and performance was evaluated at the Genus (A) and Species (B) taxonomic level. The percentage of sequences correctly classified, and percentages of false positives and false negatives are displayed. We chose a confidence threshold of 0.8 for classification of bat sequences as this value was deemed to be an acceptable tradeoff between the number of correctly classified sequences and the risk of false positives.

Our reference library included 13,126 sequences representing 512 fully identified (to species or subspecies level) bat taxa. An additional 707 sequences identified only to genus were used for genus-level classification tests. Using the RDP Classifier and BLAST (in QIIME; top match identity), we evaluated the proportion of correctly classified sequences, false positives, and false negatives at the genus and species taxonomic levels as well as the effect of the confidence threshold selected for the RDP Classifier (Fig 4) for sequences in our reference library. While taxonomic classification with BLAST correctly classified high percentages of the reference sequences, the false positive rate was higher than for the RDP Classifier (S3 Table). We therefore chose RDP Classifier for identifying sequences generated from fecal samples, as this method is conservative and performed well with guano. We selected 0.8 as our confidence threshold because this was the threshold at which field-verified bats represented in fecal samples were correctly classified while spurious classifications were minimized. The confidence threshold should be modified for each study according to the importance of false positives vs. failure to detect a species. Classification of sequences from fecal samples may improve with representation of additional bat species in databases and enhanced curation of existing databases.

#### Pooled fecal samples: Next-generation UT amplicon sequencing

**Fig 5:**
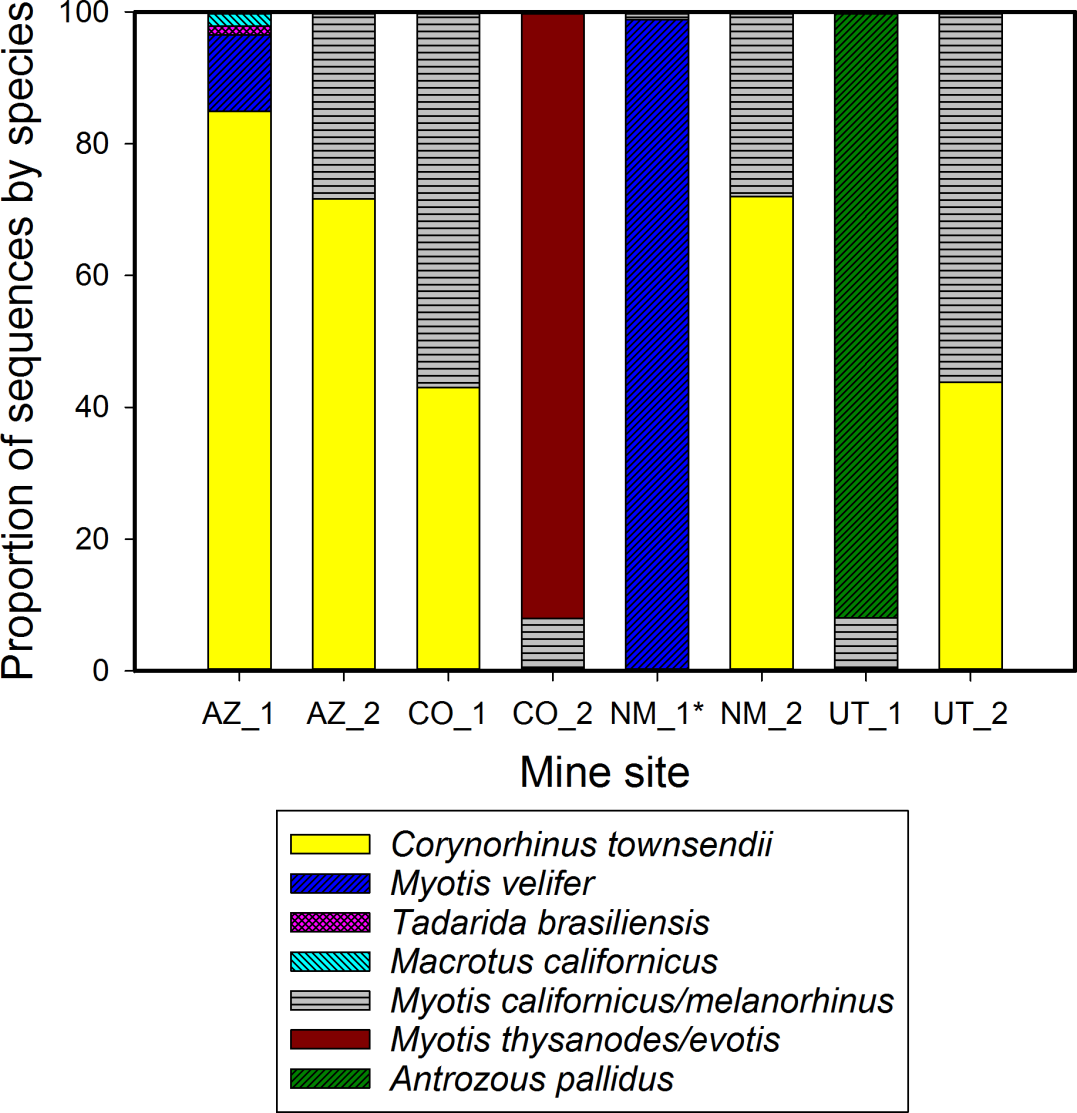
Multiple bat species detected in each of eight mines in the U.S. Southwest from next-generation amplicon sequencing of pooled guano. A single DNA extraction was performed on a pooled sample of roughly 200 fecal pellets that were collected from each mine, and the Species from Feces primer pair SFF_145f/SFF_351r was applied with next generation amplicon sequencing on an Illumina MiSeq. *Mine sample NM_1 also contained small proportions of *Corynorhinus townsendii* and *Myotis thysanodes*/*evotis*.

We detected multiple bat species (range 2–4) in all eight mines (Fig 5) by subjecting pooled guano collected across each site to universal tail amplicon sequencing (SRA accession SRP076618), and did not detect any sequences belonging to the four classes of Arthropoda. Each sample had over 10,000 high quality reads (appropriate length, no ambiguous characters, not chimeric; average Q Score = 33, average GC content = 48%). Seven species were detected: *Corynorhinus townsendii* and *Myotis californicus*/*melanorhinus* were each found in six mines, and *Macrotus californicus*, *Antrozous pallidus*, *Tadarida brasiliensis*, *Myotis velifer*, and *Myotis thysanodes*/*evotis* were each detected in 1–2 mines. In comparison, at the time of sample collection bats were only seen in three of the mines, and totaled three species [70]. Performing four DNA extractions from the same fecal slurry instead of a single extraction increased the number of reads (N = 5, W = -15, *P* ≤ 0.05, Wilcoxon signed-rank test) but not the number of bat species detected (N = 5, tied). Relative proportions of sequences do not necessarily reflect proportions of bat feces in the sample or proportion of species in the roost. We are currently testing this relationship; preliminary results showed that species represented by less fecal DNA (1 μL fecal DNA extract in 191 μL of two other species) in a sample had fewer reads than species with fecal DNA in higher proportion.

## Discussion

Together, bioinformatics and next-generation sequencing technologies can generate tools with the capacity for impressively accurate species identification within diverse taxonomic groups, fueled by readily available sample types such as feces. Our DNA mini-barcode assay proved highly successful at identifying bats to species from feces and thus provides an important new conservation tool for Chiroptera. In addition, our approach to creating this assay for one order can be replicated for other diverse taxonomic groups. The Species from Feces assay can identify 92% of bat species in our reference database, which represents roughly one-third of the bat species in the world. While >90% species-level discrimination has been achieved at higher taxonomic levels with the full 658 bp barcode (fish [71, 72]; Holarctic amphibians [73]; North American birds [74]), such broad order-wide species priming coverage with a single DNA mini-barcode is exceptional, success that we attribute to advancements in microbial genomics and associated analytical tools. The global utility of the assay will improve further as additional vouchered bat species are barcoded and sequences from private projects are made publically accessible on BOLD. These promising results successfully translated into practice with samples from 54 bat species in the lab. In no instance did a particular bat species that we tested fail to PCR amplify with our primers, our overall amplification success for singular fecal pellets was high (100%), and of the thousands of singular and pooled fecal pellets we have analyzed (this study; [70]; Fofanov *et al*., unpublished), prey or environmental arthropods were never detected, demonstrating the sensitivity and specificity of the assay. We successfully validated our system from feces of insectivorous, frugivorous, and nectarivorous bats, with fresh and 3 month old fecal pellets, and with individual and pooled guano pellets. We are currently quantifying the probability of detecting DNA of a rare species in a pool of guano DNA extract in a controlled experiment, which will be described in a subsequent paper. Results from 1:192 mixtures (1 portion of a ‘rare’ species with 191 portions split between two ‘common’ species) show 100% sensitivity and specificity across tests. We are also determining the effects of time and humidity on DNA recovery from guano, and assessing sensitivity for eDNA applications (bat DNA in water and soil).

Importantly, our Species from Feces assay entails a short amplicon that is scalable, and thus can be used efficiently in high-throughput systems such as the Illumina MiSeq as well as traditional Sanger sequencing. This means that regardless of the sequencing technology of a genetics lab, this assay is transferable and will not require the time and cost involved with developing new assays for regional species. Further, with next-generation amplicon sequencing, Species from Feces can be expanded to incorporate additional primers with little extra time, effort, or cost. It is possible to simultaneously screen for problematic taxa, such as taxa that share this DNA mini-barcode, by adding additional markers. Alternatively, barcodes for other organisms can be simultaneously screened; for instance, we are examining microbial communities in guano in addition to identifying the fecal donors (Fofanov *et al*., unpublished).

The utility of our approach is that all species that contributed to a collection of guano–be it sampled from a roost such as a cave, mine, bridge, tree hollow, bat box, building, or other type of habitat–can be efficiently and effectively determined from a single pooled sample. In this study we rapidly and accurately assigned taxonomy via the naïve Bayesian classifier to large batches of DNA mini-barcode sequences, complementing other studies that have illustrated the utility of the approach with bacteria [45], fungi [75], and insects [47]. Our approach accurately classified COI barcode sequences to the genus (~96%) or species level (~91%) when using a complete reference database and the RDP Classifier (confidence threshold of 0.8). Porter *et al*. [47], using the same classifier for insects, found that barcodes 200 bp or greater yielded similarly accurate taxonomic assignments as full length COI barcodes, a result echoed by other animal COI mini-barcode studies. Our approach provides a method for analyzing millions of Illumina reads representing thousands of guano samples in a timely manner. As for cost, we screened this single gene target for USD $50/sample in reagents and consumables. As each pooled sample contained about 200 pellets, this works out to lab costs of 25 cents per fecal pellet.

Applications are numerous and emerging. First, the Species from Feces assay can be used to assess species assemblages within roosts. For example, we have employed the assay to determine how gating mines impacts the bat species assemblages that use them [70]. Second, it can be used in conservation applications to search for species at risk such as IUCN Red List Near Threatened, Vulnerable, Endangered, and Critically Endangered species despite low densities of these rare bats. Our method can also locate previously unidentified roosts of at risk species. Third, field identifications of captured bat species that are difficult to distinguish can be confirmed by using a fecal pellet, buccal swab, or wing swab. We inadvertently found that visual identification of bats captured by mist-netting did not match genetic identification using our DNA mini-barcode for three pairs of morphologically similar species. *Myotis auriculus* was misidentified in the field as *M*. *evotis*, *Corynorhinus townsendii* as *Idionycteris phyllotis*, and *Myotis septentrionalis* as *M*. *sodalis*. These genetic identifications were later corroborated by photos taken at the time of collection, or fieldworkers were confirmed novices at identification. We recognize that species misidentification is just one potential source of error in a research pipeline, but with the ongoing spread of White-Nose Syndrome across the U.S. and Canada [76, 77] (current distribution map: https://www.whitenosesyndrome.org/), it may become important to genetically verify field identifications of bat species. Our ability to successfully employ the same wing swab DNA extraction that was used to test for the fungus means less stress for bats and no additional work for field teams. Forth, the assay can be used for novel applications such as identifying species in bat guano fertilizer. Using amplicon sequencing, we identified Yinpterochiropteran (*Eonycteris spelaea*, *Rousettus leschenaultii*) and Yangochiropteran (*Chaerephon plicatus*, *Myotis velifer*, *Tadarida brasiliensis*) species in fertilizer samples of unknown age or bat composition.

The searchable database on our website allows users to determine the capability of the assay for identifying species of interest. Searches will yield a successful prognosis, or a statement that the species has not yet been barcoded. The latter will become less of an issue as more species are barcoded and sequences are made freely-available on public databases. Non-barcoded species for which the Species from Feces assay is desired can be easily COI barcoded with positively identified reference tissue and then added to open source databases such as GenBank, BOLD, and local DNA libraries for more rapid assay application. Where species share the same DNA mini-barcode with another species, as is the case for three pairs of *Myotis* species in the southwestern U.S., primers can be tailored to target genetic variation in other genes that differentiate the species, and these can be used singly in Sanger sequencing or added as a second gene target for amplicon sequencing. For some species, the Species from Feces sequence is discriminatory, but primers are not predicted to hybridize effectively to template DNA according to parameters for *in silico* mismatch allowance; in these instances, the same primers may still favor priming competitively to bat sequences over background DNA in the laboratory, or they may be redesigned or optimized.

Genetic regions used for taxonomic identification have limitations; they may not be discriminatory for recently diverged species, in which case more than one genetic marker is required, and observed diversity may be due to unrecognized taxa or to processes such as introgression through past hybridization or incomplete lineage sorting [78, 79]. The strength of our Species from Feces DNA mini-barcode is matching an environmental sample (fecal, swab) to a positively-identified reference sequence, not definitively discovering cryptic species. However, in geographic regions with high bat species diversity and cryptic species, the Species from Feces assay will serve as a timely technological improvement to help ongoing efforts in documenting new species by allowing identification to genus or family. We base this argument on its broad taxonomic affinity (Fig 2) compared to standard PCR primers already used in such endeavors. Indeed, DNA barcoding has been found to be useful for identifying genetic units for further examination; any putative new species require the full multidimensional complement of taxonomic validation [80]. For instance, Guyanese bats and North American birds exhibited significant divergences in DNA barcodes within about 10% of species, suggesting cryptic species or divergent populations [32, 81]. As with other studies [72], the greatest challenge for Species from Feces is likely initial specimen misidentification and resulting erroneous voucher sequences; increased rigor in sequence submission criteria by public databases, in combination with greater use of bat barcodes, will help to alleviate this issue and identify suspect sequences.

### Conclusions

The evolution of genetic approaches employing DNA barcodes has resulted in taxonomic identification being practical, inexpensive, rapidly sharable, and achievable with fecal-sourced DNA. Kress *et al*. [82] suggested that the next major advancement for DNA barcoding for ecology and conservation will involve new methods of generating and analyzing DNA barcode sequences. Here, we applied recently developed methods in microbial bioinformatics and genomics to a scientifically intractable order of mammals, with high specificity, resolution, and success. This pathway can be applied to other taxa, and will become increasingly powerful to the non-microbial disciplines as reference databases are populated with publically-available barcode sequences.

## Supporting Information

S1 FASTA DNA mini-barcode sequences for 186 lab-tested genetic samples of 54 bat species. (TXT)

S2 FASTA COI sequences trimmed to each Species from Feces DNA mini-barcode (SFF_145f and SFF_351r), for 430 bat species. These sequences were BLAST searched manually against NCBI and also using the BOLD identification tool (Fig 2). They were scored based on the top five results with possible outcomes being correct species assignment, ambiguous alignment to other bat taxa, or incorrect taxonomic assignment. Each sequence header contains an associated BOLD sequence ID or a GenBank accession number.
(TXT)

S3 FASTA COI sequences representing Chiroptera, Arachnida, and Insecta used for bat DNA mini-barcode primer design. (FASTA)

S1 Protocol PCR conditions for bat barcoding primers BEGLCOIf and BEGLCOIr (580BP). (PDF)

S1 Table *In silico* performance of bat-specific DNA mini-barcode primer pairs against all publically accessible Chiroptera COI barcode sequences. In order to compare the potential of primers developed in current study to those previously utilized, we harvested sequences from the Barcode of Life Data Systems (www.barcodinglife.org) without applying the strict taxonomic parameters used for primer design, dereplicated them at full length using USEARCH (derep_fulllength), and then clustered to 100% similarity (cluster_fast) to alleviate sequence overrepresentation. All tested primers were run through PrimerProspector’s analyze_primers.py script against all unaligned sequences using default parameters, which allows for some primer mismatch. We then generated *in silico* amplicons (get_amplicon_and_reads.py), and visually inspected and removed all amplicon files for long N-characters and appropriate size. The total number of sequences for bats is in parentheses, with the number of hits for each primer pair below.
(PDF)

S2 Table *In vitro* comparison of bat-specific DNA mini-barcode primer pairs (products <300BP) for use with fecal DNA. PCR amplification adhered to the conditions outlined in the respective primer publications. All tests were assessed by amplification performance to Sanger sequencing as well as ability to identify species-level taxonomy using common DNA alignment-based identifiers. DNA was isolated from eight singular fecal pellets and one tissue sample for a test panel that included five bat species of two families. Species tested were *Eptesicus fuscus* (one fresh fecal pellet and four at room temperature for three months), *Myotis auriculus*, *Corynorhinus townsendii*, *Tadarida brasiliensis*, and *Euderma maculatum* (internal tissue). The total number of samples are in parentheses.
(PDF)

S3 Table Evaluation of taxonomic classification of reference library sequences by BLAST and RDP Classifier. The primer-targeted regions of reference library sequences were evaluated as six datasets: unique amplicon regions against the full reference library, and five random subsamples of the reference library (see Methods). Classifications with the RDP Classifier were computed with confidence thresholds of 0.5–1.0, and performance was evaluated at the Genus and Species taxonomic level. While taxonomic classification with BLAST correctly classified high percentages of the reference sequences, the false positive rate was higher than for the RDP Classifier. We therefore chose RDP Classifier for identifying sequences generated from fecal samples, and selected a confidence threshold of 0.8 because this was an acceptable tradeoff between the number of correctly classified sequences and the risk of false positives.
(XLSX)

S4 Table GenBank accession numbers for 186 Sanger-derived bat COI mini-barcode sequences in Table 2. (XLSX)
