# Supplementary material for: Species From Feces: Order-Wide Identification of Chiroptera From Guano and Other Non-Invasive Genetic Samples
Source: PLoS One. 2016 Sep 21;11(9):e0162342. doi: 10.1371/journal.pone.0162342 (PMC5031397; doi:10.1371/journal.pone.0162342)
Supplement: S1 Protocol — (PDF) [file pone.0162342.s004.pdf]

S1 Protocol. PCR conditions for bat barcoding primers BEGLCOIf and BEGLCOIr (580BP). Barcode amplification reactions included 0.5-2 ng DNA in a 25 µL volume. For low quality DNA samples deriving from buccal, fecal, or wing punch sources, DNA was added undiluted. The reaction included 1 µL 10X Mg-free PCR buffer (Invitrogen), 2 mM $MgCl_2$, 20 ng BSA, 0.2 mM of each dNTP, 0.4 µM unlabeled primers, and 0.05 (tissue) - 0.1 (feces) U/ µL PlatinumTaq DNA polymerase (Invitrogen). Amplification was carried out on a PTC-200 thermocycler under the following conditions: 95°C for 10 minutes followed by 33 cycles of 95°C for 30s, 59°C for 30s, and 72°C for 1 min, with a final extension step at 72°C for 10 min. Products were purified using ExoSAP-IT (Affymetrix), cycle-sequenced using BigDye Terminator v3.1 (manufacturer instructions), and sequenced on an ABI3130 Genetic Analyzer (applied Biosystems). Sequences were then visualized and processed using Sequencher 5.3 software.
