# Supplementary material for: Species From Feces: Order-Wide Identification of Chiroptera From Guano and Other Non-Invasive Genetic Samples
Source: PLoS One. 2016 Sep 21;11(9):e0162342. doi: 10.1371/journal.pone.0162342 (PMC5031397; doi:10.1371/journal.pone.0162342)
Supplement: S1 Table — In order to compare the potential of primers developed in current study to those previously utilized, we harvested sequences from the Barcode of Life Data Systems (www.barcodinglife.org) without applying the strict taxonomic parameters used for primer design, dereplicated them at full length using USEARCH (derep_fulllength), and then clustered to 100% similarity (cluster_fast) to alleviate sequence overrepresentation. All tested primers were run through PrimerProspector’s analyze_primers.py script against all unaligned sequences using default parameters, which allows for some primer mismatch. We then generated in silico amplicons (get_amplicon_and_reads.py), and visually inspected and removed all amplicon files for long N-characters and appropriate size. The total number of sequences for bats is in parentheses, with the number of hits for each primer pair below. (PDF) [file pone.0162342.s005.pdf]

S1 Table. ***In silico*** **performance of bat-specific DNA mini-barcode primer pairs against all publically accessible Chiroptera COI barcode sequences.** In order to compare the potential of primers developed in current study to those previously utilized, we harvested sequences from the Barcode of Life Data Systems (www.barcodinglife.org) without applying the strict taxonomic parameters used for primer design, dereplicated them at full length using USEARCH (derep_fulllength), and then clustered to 100% similarity (cluster_fast) to alleviate sequence overrepresentation. All tested primers were run through Primer Prospector's analyze_primers.py script against all unaligned sequences using default parameters, which allows for some primer mismatch. We then generated *in silico* amplicons (get_amplicon_and_reads.py), and visually inspected and removed all amplicon files for long N-characters and appropriate size. The total number of sequences for bats is in parentheses, with the number of hits for each primer pair below.

Primer	Author	Comparable *in silico*?	Bat sequences (10523)
VF1<br>BC1R	Ivanova et al. 2006<br>Ivanova et al. 2012	No*	38
BC2F<br>BC2R	Ivanova et al. 2012<br>Ivanova et al. 2012	Yes	1182
BC3F<br>BC3R	Ivanova et al. 2012<br>Ivanova et al. 2012	Yes	805
BC4F<br>BC4R	Ivanova et al. 2012<br>Ivanova et al. 2012	Yes	0
BC5F<br>BC5R	Ivanova et al. 2012<br>Ivanova et al. 2012	Yes	0
BC6F<br>VR1	Ivanova et al. 2012<br>Ward et al. 2005	No*	5
SFF_145f<br>SFF_351r	Walker et al. 2016<br>Walker et al. 2016	Yes	6088
SFF_145f<br>SFF_492r	Walker et al. 2016<br>Walker et al. 2016	Yes	6988
SFF_210f<br>SFF_492r	Walker et al. 2016<br>Walker et al. 2016	Yes	5726
SFF_348f<br>SFF_492r	Walker et al. 2016<br>Walker et al. 2016	Yes	4958
SFF_210f<br>SFF_351r	Walker et al. 2016<br>Walker et al. 2016	Yes	5124
SFF_145f<br>SFF_348r	Walker et al. 2016<br>Walker et al. 2016	Yes	9126
SFF_210f<br>SFF_348r	Walker et al. 2016<br>Walker et al. 2016	Yes	5124
SFF_351f<br>SFF_492r	Walker et al. 2016<br>Walker et al. 2016	Yes	5759
BEGLCOIf<br>BEGLCOIr	Walker et al. 2016<br>Walker et al. 2016	Yes	7618

*Primers hybridize outside of the standard barcode region, and thus are not strictly comparable using the downloaded sequence dataset.
