# Supplementary material for: Species From Feces: Order-Wide Identification of Chiroptera From Guano and Other Non-Invasive Genetic Samples
Source: PLoS One. 2016 Sep 21;11(9):e0162342. doi: 10.1371/journal.pone.0162342 (PMC5031397; doi:10.1371/journal.pone.0162342)
Supplement: S2 Table — PCR amplification adhered to the conditions outlined in the respective primer publications. All tests were assessed by amplification performance to Sanger sequencing as well as ability to identify species-level taxonomy using common DNA alignment-based identifiers. DNA was isolated from eight singular fecal pellets and one tissue sample for a test panel that included five bat species of two families. Species tested were Eptesicus fuscus (one fresh fecal pellet and four at room temperature for three months), Myotis auriculus, Corynorhinus townsendii, Tadarida brasiliensis, and Euderma maculatum (internal tissue). The total number of samples are in parentheses. (PDF) [file pone.0162342.s006.pdf]

S2 Table. ***In vitro* comparison of bat-specific DNA mini-barcode primer pairs (products <300BP) for use with fecal DNA**. PCR amplification adhered to the conditions outlined in the respective primer publications. All tests were assessed by amplification performance to Sanger sequencing as well as ability to identify species-level taxonomy using common DNA alignment-based identifiers. DNA was isolated from eight singular fecal pellets and one tissue sample for a test panel that included five bat species of two families. Species tested were *Eptesicus fuscus* (one fresh fecal pellet and four at room temperature for three months), *Myotis auriculus*, *Corynorhinus townsendii*, *Tadarida brasiliensis*, and *Euderma maculatum* (internal tissue). The total number of samples are in parentheses.

Primer	Author	Amplification (of 9)	Sequencing (of 9)	BLAST ID (species-level)	BOLD ID	Performance (%)
VF1<br>BC1R	Ivanova et al. 2006<br>Ivanova et al. 2012	3	0	NA	NA	NA
BC2F<br>BC2R	Ivanova et al. 2012<br>Ivanova et al. 2012	0	NA	NA	NA	NA
BC3F<br>BC3R	Ivanova et al. 2012<br>Ivanova et al. 2012	0	NA	NA	NA	NA
BC4F<br>BC4R	Ivanova et al. 2012<br>Ivanova et al. 2012	4	0	NA	NA	NA
BC5F<br>BC5R	Ivanova et al. 2012<br>Ivanova et al. 2012	0	NA	NA	NA	NA
BC6F<br>VR1	Ivanova et al. 2012<br>Ward et al. 2005	8	8	7**	0**	78%
SFF_145f<br>SFF_351r	Walker et al. 2016<br>Walker et al. 2016	9	9	9	9	100%

** Mini-barcode marker had insufficient resolution to yield credible matches for alignment-based identification.
